# Molecular basis and therapeutic targets in prostate cancer: A comprehensive review

**DOI:** 10.17305/bb.2023.8782

**Published:** 2023-10-01

**Authors:** Florentina Claudia Militaru, Valentin Militaru, Nicolae Crisan, Ioana Corina Bocsan, Anghel Adrian Udrea, Andreea Catana, Eniko Kutasi, Mariela Sanda Militaru

**Affiliations:** 1 Department of Pharmacology, Toxicology and Clinical Pharmacology, University of Medicine and Pharmacy, Cluj-Napoca, Romania; 2 Medisprof Cancer Center, Cluj-Napoca, Romania; 3 Department of Internal Medicine, Iuliu Hatieganu University of Medicine and Pharmacy, Clinical County Hospital, Cluj-Napoca, Romania; 4 Department of Urology, Iuliu Hatieganu University of Medicine and Pharmacy, Cluj-Napoca, Romania; 5 Department of Molecular Sciences, Medical Genetics, Iuliu Hatieganu University of Medicine and Pharmacy, Cluj-Napoca, Romania; 6 Institute of Oncology I. Chiricuta, Cluj-Napoca, Romania

**Keywords:** Prostate cancer, pathways, genetic syndromes, targeted therapy

## Abstract

Prostate cancer is one of the most significant causes of morbidity and mortality in male patients. The incidence increases with age, and it is higher among African Americans. The occurrence of prostate cancer is associated with many risk factors, including genetic and hereditary predisposition. The most common genetic syndromes associated with prostate cancer risk are *BRCA*-associated hereditary breast and ovarian cancer (HBOC) and Lynch syndrome. Local-regional therapy, i.e., surgery is beneficial in early-stage prostate cancer management. Advanced and metastatic prostate cancers require systemic therapies, including hormonal inhibition, chemotherapy, and targeted agents. Most prostate cancers can be treated by targeting the androgen-receptor pathway and decreasing androgen production or binding to androgen receptors (AR). Castration-resistant prostate cancer (CRPC) usually involves the PI3K/AKT/mTOR pathway and requires targeted therapy. Specific molecular therapy can target mutated cell lines in which DNA defect repair is altered, caused by mutations of *BRCA2,* partner and localizer of *BRCA2* (*PALB2*), and phosphatase and tensin homolog (*PTEN*) or the transmembrane protease serine 2-ERG (*TMPRSS2-ERG*) fusion. Most benefits were demonstrated in cyclin-dependent-kinase 12 (*CDK12*) mutated cell lines when treated with anti-programmed cell death protein 1 (PD1) therapy. Therapies targeting *p53* and AKT are the subject of ongoing clinical trials. Many genetic defects are listed as diagnostic, prognostic, and clinically actionable markers in prostate cancer. AR splice variant 7 (AR-V7) is an important oncogenic driver and an early diagnostic and prognostic marker, as well as a therapeutic target in hormone-resistant CRPC. This review summarizes the pathophysiological mechanisms and available targeted therapies for prostate cancer.

## Introduction

Prostate cancer is the second most frequently diagnosed cancer in males, after the lung cancer, according to statistics. Furthermore, regarding mortality rates, prostate cancer seems to be the fifth cause of cancer-related deaths in males worldwide [1]. The incidence of prostate cancer appears to be correlated with older age. The average age at which males are diagnosed is 66 years. Race is also a critical consideration, as African Americans have a higher incidence of prostate cancer, while Asian males have a lower incidence than Caucasians. African Americans also have a much higher mortality rate associated with prostate cancer. Genetic factors and lifestyle might explain the differences. Dietary factors, body mass index (BMI), and physical activity must also be considered when determining the risk of developing prostate cancer [2]. Malignant tumors of the prostate can often be asymptomatic, making early diagnosis more difficult. More common symptoms are difficulty urinating or emptying the bladder, urgent urination, bloody urine, and nocturia. Sometimes it can present with lower back pain due to bone metastasis [3]. Hereditary prostate cancer is suspected if there is a family history of prostate cancer in at least three generations, either in the maternal or paternal lineage, if at least three first-degree relatives have been diagnosed with prostate cancer, if at least two relatives were diagnosed before the age of 55, or even if there are documented cases of other cancers in the family, such as breast, ovarian, or pancreatic cancer. Genetic testing is performed through next-generation sequencing (NGS), which allows whole genome or whole exome sequencing and thus finds the various genes or nucleotides which may present genetic alterations. Identifying germinal mutations can also play a part in choosing the correct treatment protocol for the patients and offering proper genetic counseling [4].

## Risk factors

Several risk factors are known to be associated with prostate cancer, some of which are either somatic or germline genetic mutations. Age is considered a major risk factor, as most prostate cancers occur after the age of 65, while diagnosis before the age of 40 is rare. Ethnicity and lifestyle also play a role in prostate cancer risk assessment. A family history of prostate cancer also increases the risk of developing malignant prostate tumors. For example, it seems that having brothers diagnosed with prostate cancer represents a higher risk than having a father who has had a disease. Obesity, smoking, exposure to toxic chemicals, vasectomies, prostatitis, and sexually transmitted infections are also considered risk factors, as studies demonstrated their correlation to higher morbidity and mortality rates [5]. Increased BMI is linked to high-grade prostate cancer and overall mortality. Obesity and metabolic syndrome induce dysregulation of the insulin axis, promote inflammatory cytokine signaling, and result in DNA-damaging, oxidative stress, and subsequent carcinogenic effect [6].

A significant proportion of prostate cancer susceptibility has been attributed to an inherited predisposition. Approximately 15%–20% of all cases occur in a hereditary, familial context and include high to moderate penetrant genes also reported as a genetic risk factor for other cancers: breast cancer gene 1 (*BRCA1*), *BRCA2*, ataxia telangiectasia mutated (*ATM*), checkpoint kinase 2 (*CHEK2*), partner and localizer of *BRCA2* (*PALB2*), mutL homolog 1 (*MLH1)*, mutS homolog 2 (*MSH2)*, *MSH6*, *PMS2*. *BRCA1* and *BRCA2* genes are mainly discussed for hereditary breast and ovarian cancers, although there is a higher risk for other malignancies like colon cancer, melanoma, thyroid cancer, and prostate cancer [7]. Germline mutations in *BRCA*, especially the *BRCA2* gene, are a well-known genetic risk factor for developing malignant prostate tumors. *BRCA1* carriers have a 1.8-fold to 3.8-fold increased relative risk (RR) of diagnosis by the age of 65. The risk is ever higher for *BRCA2* carriers, from 2.5-fold to 8.6-fold by the age of 65, more frequently with a younger onset, more aggressive phenotype, and higher mortality rates.

Lynch syndrome is also considered a risk factor for several cancer types, including prostate cancer. Lynch syndrome appears due to genetic alterations of DNA mismatch repair genes, leading to genetic material defects and a higher risk for tumor development. While this syndrome is primarily associated with gastrointestinal cancers, it also plays a part in prostate cancer. Epithelial cellular adhesion molecule gene (*EPCAM)* mutations lead to increased methylation of the *MSH2* promoter, eventually leading to MSH2 protein loss, thus resembling characteristics of Lynch syndrome and defective DNA mismatch repair [8]. The *HOX* genes play an essential role in the embryologic development of different tissues. Mutations in the *HOXB13* gene have been associated with highly increased risk of prostate cancer, although they are responsible for only a small fraction of prostate cancer cases worldwide [9]. Studies showed a correlation between *CHEK2* missense mutations and a higher risk for prostate cancer, but familial clustering had not been demonstrated. Due to its role in encoding a G2 checkpoint kinase, the *CHEK2* gene plays a crucial role in DNA repair [10]. Similarly to *BRCA2* mutations, *NBS1* gene mutations affect tumor suppression. In addition, *NBS1* mutations have been linked to immunodeficiency, chromosomal instability, and predisposition to developing cancers. Prostate cancers in which *NBS1* mutation is identified tend to be more aggressive and have a poor prognosis even when conventional therapy is administered [11].

Several somatic mutations have been linked to prostate cancer, usually indicating a higher risk of developing metastatic disease and potential resistance to treatment. Mutations in the *PIK3CA* oncogene, in which the function is associated with cell differentiation and migration, have been described in multiple human cancers, including prostate cancer. Other somatic mutations, such as those occurring in *KIT*, *BRAF*, or *TP53* genes, were correlated with cell overproliferation, more advanced stages of the disease, and a poor prognosis. While tumor cells with *BRAF* mutations may be candidates for *BRAF* inhibitors, and those with *KIT* mutations seem to respond to tyrosine kinase inhibitors, *TP53* mutated cells exhibit resistance to multiple therapies, including cisplatin, alkylating agents, antimetabolites, or anthracyclins [12–14].

A limited role in developing hereditary prostate cancer was attributed to zinc phosphodiesterase ELAC protein 2/histone promoter control protein 2 (*ELAC2/HPC2)* gene mutations. In addition, from the identified genetic variants, the Glu216Stop nonsense mutation seemed responsible for some familial prostate cancer cases [15]. Studies were performed to determine if macrophage scavenger receptor 1 (*MSR1*) mutations can be linked to hereditary prostate cancer. However, these mutations appear to have moderate penetrance and are detected in a limited number of cases, therefore, the correlation between hereditary prostate cancer and *MSR1* mutations is not currently significant [16]. RNase L gene (*RNASEL*) plays a role in cancer prevention by degrading RNA, determining cellular stress response and apoptosis. Genetic mutations in this gene lead to defective apoptosis and an increased risk of developing malignant tumors. In prostate cancer, *RNASEL* mutations are associated with younger age of onset and more aggressive course of the disease [17]. Loss of retinoblastoma 1 (*RB1*) is also an essential factor in prostate cancer. Together with *TP53*, it increases cell proliferation and decreases androgen receptor (AR) signaling. Tumors that present combined loss of *RB1* and *TP53* are very aggressive, do not respond to AR antagonist treatment and are associated with a limited survival rate. Therefore, targeted molecular therapy must be considered in these cases [18]. Genetic alterations in the *ATM* gene follow a similar mechanism to those linked with *BRCA2*, *BRCA1*, or *CHEK2* mutations. In addition, higher tumor progression rates were described in patients with *ATM* mutations and increased sensitivity to ionizing radiation as a risk factor for tumorigenesis [19]. *PALB2* mutations were associated with more aggressive forms of prostate cancer and a higher mortality rate [20]. *CDK12* inactivation, a DNA damage response gene, leads to aggressive prostate cancers that do not respond to treatment with hormone therapy, taxanes, or poly ADP-ribose polymerase inhibitors (PARPi). However, immunotherapy with programmed cell death protein 1 (PD-1) inhibitors seems effective for these patients and represents a viable option for their therapeutical management [21].

Single nucleotide polymorphisms (SNPs) are reported to have a lower to moderate effect on prostate cancer progression compared to combinations of SNPs, leading to more severe progression patterns. There are more than 100 common SNPs described in association with prostate cancer. These genetic alterations involve multiple mechanisms leading to tumor progressions, such as oxidative stress or impaired steroid metabolism, defective DNA repair, increased angiogenesis, and cell adhesion. In addition, SNPs can determine a higher risk of relapsing in prostate cancer patients treated with androgen deprivation therapy. Correctly identifying SNPs, alongside somatic and germline gene mutations, and knowing the pathophysiological mechanism they imply, represent the future of precision medicine and individualized targeted treatment options [22].

**Figure 1.**
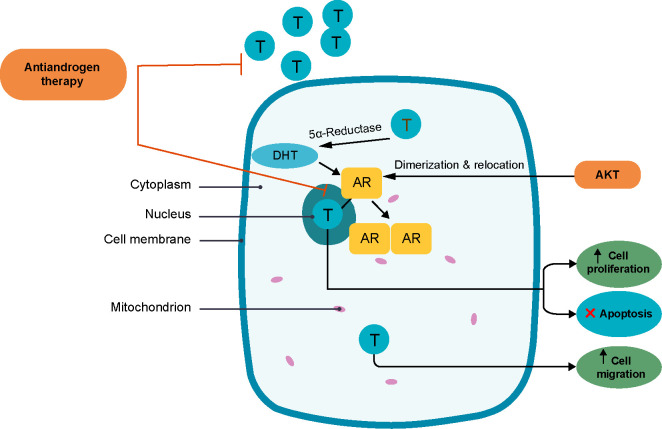
**Androgen receptor pathway [24].** DHT: Dihydrotestosterone; T: Testosterone; AR: Androgene receptor.

## Pathophysiology of prostate cancer

While metabolism alterations are not considered a cause of malignant lesions, they promote tumor cell survival and proliferation. Typically, cells rely on aerobic pathways to convert metabolites into the energy they need, but tumor cells use anaerobic pathways. Thus, glycolysis is less efficient, but ATP is produced faster. Increased glucose intake leads to increased production of endogenous fatty acids, which is not usually observed in healthy cells. Most tumor cells express higher levels of Acetyl-CoA carboxylase, fatty acid synthase (FASN), and lipogenic enzymes. Tumors with increased FASN levels lead to poor prognosis. Treating cancer cells with cerulenin, which inhibits FASN, showed promising effects, as it inhibited cell proliferation and even induced cell death [23].

The AR pathway is the most frequently responsible for tumorigenesis and tumor growth in prostate cancers. Testosterone and 5α-dihydrotestosterone are crucial in tumor development and progression. When entering the nucleus, sex steroids bind to promoter regions of genes, regulate cell proliferation, and provide the means to escape apoptosis. Sex steroids are also involved in cell cycle control and migration by binding to molecules outside the nucleus. The AKT serine/threonine kinase plays a role in the dimerization and relocation of AR on the nucleus membrane (Figure 1). Approximately 80%–90% of malignant tumors in the prostate depend on androgens for their development, which is why anti-androgen therapy represents an essential pillar in prostate cancer management. Some prostate cancers, called castration-resistant prostate cancers (CRPC), will not respond to androgen ablation therapy. These tumor cells seem to have an intrinsic androgens production [24].

AR splice variant 7 (AR-V7) genetic alterations affect the *AR* gene located on the X chromosome and produce splice-variant ARs. Although results are debatable, recent studies have shown more aggressive growth patterns and decreased overall survival in patients with AR-V7 mutations. Correlations have also been demonstrated between AR-V7 mutations, abiraterone, and enzalutamide resistance. Thus, the AR-V7 mutation status can be a prognostic marker for metastatic CRPC [25]. Non-coding RNAs have essential regulatory roles in different molecular pathway levels, such as transcription and translation. They are involved in malignant cell proliferation, decreased apoptosis, metastasis formation, and drug resistance. In prostate cancer, they regulate AR activity. Because they are characterized by high stability in biological fluids, non-coding RNAs may be good candidates for markers in liquid biopsies. Targeting them as a therapeutic approach could inhibit AR expression and the disease progression [26, 27].

**Figure 2.**
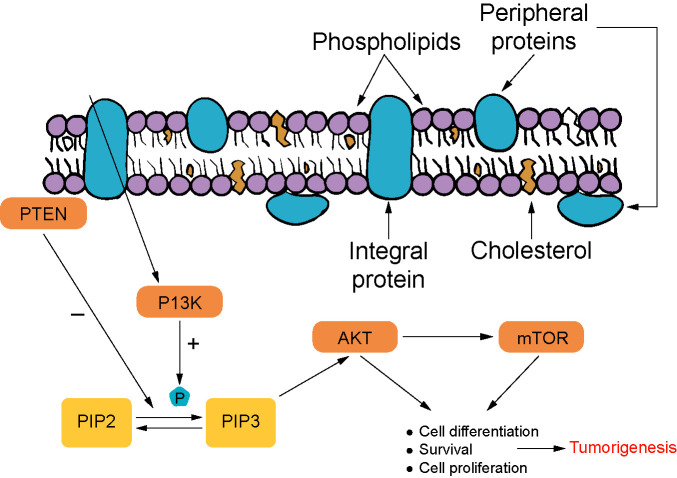
**PI3K pathway in tumorigenesis [28, 29].** PTEN: Phosphatase and tensin homolog; PI3K: Phosphatidylinositol 3-kinase; mTOR: Mechanistic target of rapamycin; PIP3: Phosphatidylinositol-3,4,5-triphosphate.

Phosphatidylinositol 3-kinase (PI3K) signaling is responsible for most cases in which prostate cancers are castration-resistant. The PI3K pathways are associated with more aggressive tumor growth and poor outcome. This pathway plays a part in obtaining phosphatidylinositol-3,4,5-triphosphate (PIP3) and AKT activation. PIP3 formation is inhibited by phosphatase and tensin homolog (PTEN). This step is strictly linked to PTEN’s binding to the plasma membrane. Out of the advanced cases of prostate cancer, 70%–100% show aberrations in PI3K/AKT/mTOR signaling pathways. Loss of PTEN, typically membrane-localized, leads to overactivation of PI3K pathway and, therefore, tumorigenesis in the prostate glandular tissue. Inhibiting AKT signaling shows promising results in stopping tumor formation and growth and can result in tumor shrinkage. The PI3K pathway is widely involved in castration-resistant tumor pathophysiology and interacts with androgen signaling (Figure 2) [28, 29].

Autophagy is a complex process affecting cancer cells, as it promotes both cell death in the early stages of tumor development and cancer-cell proliferation once the mass is formed. There are three main types of autophagy: chaperone-mediated autophagy (CMA), microautophagy, and the primary form, macroautophagy. The last one plays a significant role in cellular homeostasis [30]. The autophagy mechanism has different phases from initiation to elongation and, finally, fusion with the lysosome, implying that various molecular pathways are involved. 5’AMP adenosine monophosphate-activated protein kinase (AMPK), mechanistic target of rapamycin (mTOR), and ATGs are the most well-known regulators of autophagy [31]. The mechanism has yet to be entirely understood, but a particular role is attributed to the PI3K/AKT/mTOR signaling pathway. PTEN and AMPK downregulate the pathway and promote autophagy. Some data showed that autophagy could be associated with chemotherapy resistance of prostate cancer cells. Further studies are needed to determine whether autophagy modulators can represent a therapeutic option for prostate cancer and how to use this complex process for the patient’s benefit [32].

**Figure 3.**
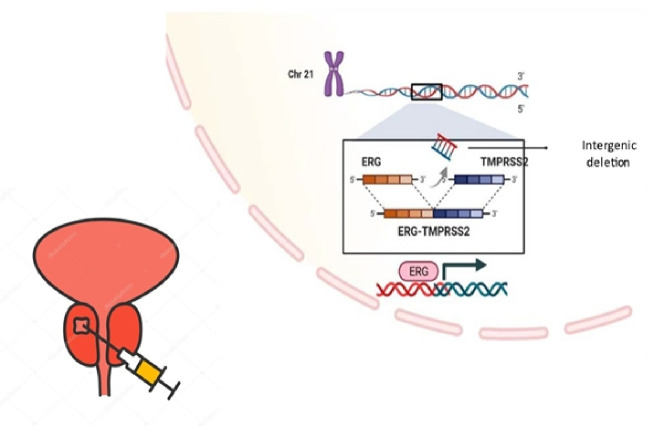
**Role of *TMPRSS2-ERG* gene fusion in prostate cancer [33, 34].** TMPRSS2: Transmembrane protease serine 2.

Transmembrane serine protease 2 (TMPRSS2) is a transmembrane protein primarily found in prostate’s secretory epithelium, prostate cancer cells, and in pancreatic and colon cancer specimens. *TMPRSS2-ERG* gene fusions can diagnose prostate cancer on a molecular level. Tumors that express *TMPRSS2-ERG* gene fusions are considered fusion-positive prostate cancers. These tumors present genetic alterations with oncogene overexpression and inactivation of tumor suppressor genes. The ERG protein has been proven to have a role in regulating the AR pathway. Research shows that ERG overexpression is more significant in the peripheral zone of the prostate (Figure 3). *TMPRSS2-ERG* fusions are responsible for checkpoint impairment in the cell cycle and for tumor proliferation, but also tumor cell migration and invasiveness by overexpression on metalloproteinase 9 (MMP9) and plexin A2. The gene fusion affects chromosome 21 and seems most frequent in the Indian and Caucasian populations [33, 34].

Iron homeostasis also plays an essential role in prostate cancer. Increasing transferrin receptor protein 1 (TfR1) levels and progressive ferroportin downregulation have been associated with transforming primary tumors to metastatic cancer in prostate cancer [35]. While physiologic iron levels favor the proliferation of prostate cancer cells, excess iron causes deleterious reactive oxygen species (ROS) to increase, subsequently activating cell death programs. Interestingly, tumor microenvironment (TME)-associated macrophages loaded with iron seem to have a pro-inflammatory effect on tumor cells [36], and propose iron supplementation as a potential targeted treatment for prostate cancer [37].

More recent studies showed that viruses linked to respiratory infections, including SARS-CoV-2, need the *TMPRSS2-ERG* complex to enter cells, and it has been discussed if prostate cancer patients are more exposed to these infections due to *TMPRSS2-ERG* overexpression. However, gene fusion represents an essential diagnostic tool and a potential therapy target [38, 39].

As the most critical TME component, cancer-associated fibroblasts (CAFs) influence tumorigenesis and impact metastasizing and therapeutic resistance for prostate cancer. CAFs might be used as a prognostic factor. CAFs are prostate cancer’s major cellular stromal component and promote cancer progression through lactate metabolism. The alteration of NAD^+^/NADH ratio through lactate uptake, further sirtuin 1 (SIRT1)-dependent progastricsin (PGC)-1α activation, and subsequent excessive mitochondria activity results in oncometabolite superoxide accumulation [40]. Further studies are yet needed to determine to what extent therapy can target CAFs to stop tumor growth and extension [41].

Long non-coding RNAs could play an essential role in prostate cancer pathogenesis [42]. Dysregulation of long non-coding RNA leads to either a tumor suppressor or oncogenic effect in cancerous cells and influences tumor proliferation and metastasis. For example, growth arrest specific 5 (*GAS5*), a potent tumor inhibitor, interferes with AKT/mTOR signaling pathway by targeting microRNA mir-103. Maternally expressed 3 (*MEG3*), another tumor-inhibiting factor, inhibits the sponge miR-9-5p cycle and induces gene silencing. On the other hand, tumor growth promoters like prostate cancer-associated transcript 1 (PCAT1), PVT1, and urothelial cancer associated 1 (UCA1) act as transcriptional repressors, a mir-145-5P sponge, and a P13K/AKT pathway activator [43].

**Figure 4.**
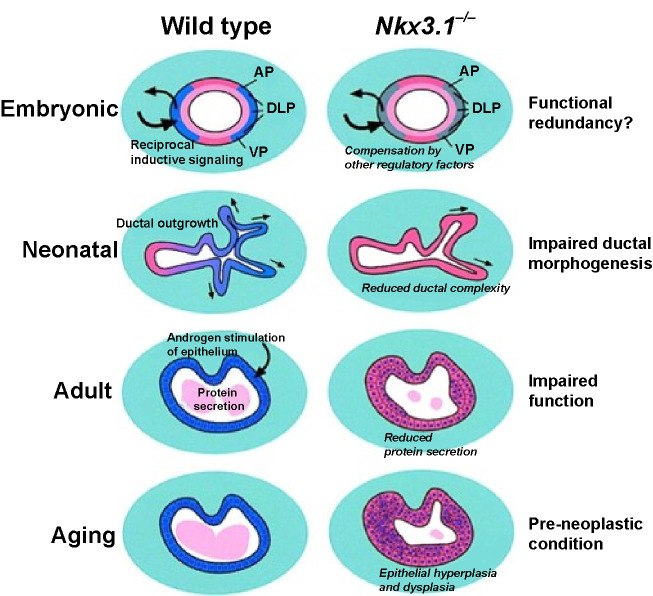
**Effects of NKX3.1 loss in tissue development and tumorigenesis [44].** NKX3.1: NK3 homeobox 1.

NK3 homeobox 1 (NKX3.1) protein is normally expressed in healthy prostate cells. In mice, NKX3.1 has growth-suppressing and differentiation-promoting effects, but its role as a tumor suppressor could not be demonstrated in human prostate cancers. With disease severity, NKX3.1 levels are decreasing. Complete loss can be documented in most metastatic prostate cancers. This phenomenon might be explained by the NKX3.1 protein being expressed only by normal cells of the prostate. The most critical role of the protein is currently as a prognostic marker. However, it might be a helpful tool for developing targeted therapies that are tissue-specific to treat the malignant tumors of the prostate and prevent as many side effects as possible (Figure 4) [44].

Malignant prostate tumors are biochemically characterized by much-decreased citrate and zinc levels compared to unaffected prostate tissue. This fact might be explained by the inability of malignant cells to accumulate and deposit zinc due to partial or total loss of the genes which encode the zinc uptake transporter. Restoring zinc levels can be an essential prophylactic or even therapeutic measure in the approach to prostate cancer. Usually, zinc levels are high, especially in the peripheral zone of the prostate. Since zinc is vital for citrate production, the citrate levels will also be low. This biochemical manifestation often occurs in the premalignant stages before the development of malignant tumors. Studies did not show that dietary zinc supplements help prevent prostate cancers [45].

## Screening and prevention

Correctly implemented screening protocols are an important tool in cancer management, as they can help identify cancers early when patients can benefit the most from the treatment. Nevertheless, as the psychological impact of a cancer diagnosis is significant, it is essential to choose wisely the screening methods and their target groups. Regarding prostate cancer, the issue is delicate, as not all prostate cancers require treatment [46].

The currently used screening method consists of prostate-specific antigen (PSA) level testing. Magnetic resonance imaging (MRI) testing is not feasible for the whole population of male patients who might be at risk. In addition, the most significant limitation of PSA testing is that it cannot discriminate between aggressive cancers and clinically indolent tumors. It is still discussed if men who are not considered at higher risk should undergo screening for prostate cancer, but it is established that those with a life expectancy lower than 10 years do not benefit from PSA-level screening [47].

Screening is recommended for people with a family history of prostate cancers, African Americans, and those with documented germline mutations in the *BRCA2* gene, starting at 40–45 years of age, depending on each guideline. *BRCA1* mutations are starting to be considered as an indication for screening at a younger age. However, more genetic alterations should be included in the guidelines in the near future to offer proper care for these patients [48].

Although studies were conducted on prostate cancer prevention, there were not any clear guidelines about active steps that can be taken to prevent it from occurring. However, some studies showed that besides having a healthy, balanced lifestyle and avoiding toxic exposure, specific diets and vitamin supplements can either increase or decrease the risk of developing prostate cancer. A slight increase in risk has been described for people whose diets include lots of dairy foods and calcium. Vitamin E and folic acid seem also to increase the risk of developing prostate cancer. Opposite to folic acid, folate has been listed as a protective factor against malignant prostate tumors [49].

As most prostate cancers rely on androgens for tumor growth and proliferation, drugs that lower testosterone levels, such as finasteride and dutasteride, lower the risk of developing malignant prostate tumors. However, a medication that lowers testosterone and thus 5α-dihydrotestosterone causes side effects that affect the patient’s quality of life, such as lower libido, gynecomastia, or erectile dysfunction. Therefore, considering all the facts, administering the medication as a primary prophylactic measure is not part of the clinical practice. Furthermore, it remains unclear if finasteride and dutasteride also lower mortality if the patients develop prostate cancers [50].

## Diagnostic strategies

If high PSA or prostate cancer antigen 3 gene (PCA3), levels are found during the screening for prostate cancer, or if the clinical rectal examination raises any suspicions, a core needle biopsy is recommended as a diagnostic method. The biopsy can be either transrectal or transperineal and samples should be collected from different prostate parts. The procedure is guided by transrectal ultrasound (TRUS) or MRI. The standard systematic diagnostic biopsy of the prostate using TRUS is slowly being replaced by MRI-based techniques. In-bore MRI-guided biopsy provides high-quality enhanced images for detection and biopsy guidance but requires MR-compatible facilities and limited access [51]. Cognitive TRUS-targeted biopsy requires a two-step procedure with the initial MRI localization of the tumor followed by an ultrasound-guided biopsy. Multiparametric MRI and fusion-guided biopsy have demonstrated clinical benefits over systematic biopsy alone, leading to improved diagnosis, risk stratification, and treatment [52].

The urologist and morphopathologist are important in the multidisciplinary team, ensuring correct patient management [53]. For a complete diagnosis, calculating the Gleason score is necessary. Based on the cellular atypia grade described during the morphophonological examination, a score is assigned to the two most dominant cell types, from one to five, in which one represents little or no cellular atypia and five highly atypical prostate cells. The sum of the two established grades represents the Gleason score. A score above seven associates a high risk for cancer development and aggressive spreading. Thus, the Gleason score also indicates the prognosis of the patient [54]. If prostate cancer is diagnosed, it is essential to determine whether metastasis exists. Potential metastasis most commonly affects the lymph nodes, bones, lungs, and liver. It can be diagnosed using CT scans, MRIs, or PET scans. Prostate-specific membrane antigen (PSMA)-PET scan can also detect prostate cancer cells in case of biochemical recurrence, using the radioactive properties of PSMA [55].

Liquid biopsy is a powerful, minimally invasive tool to detect clinically actionable molecular targets. Still, there are many challenges to overcome until liquid biopsies are implemented as routine analysis in screening and early diagnosis of prostate cancer. The multi-cancer early detection test (GRAIL) study based on Circulating Cell-free Genome Atlas (CCGA) [56] validated a pan-cancer methylation targeted-based assay, among others, in prostate cancer early diagnosis. The test showed an excellent overall sensitivity of early detection, however, detecting prostate cancer using the multi-cancer assay was lower and similar to other screening programs.

## Treatment

There are several treatment options for prostate cancer, but not all require aggressive approaches, so choosing the proper management is essential. In addition, it must be considered whether the patient is symptomatic, how advanced the cancer is, the life expectancy, and the quality of life of the patient, and whether the patient has important comorbidities [57]. Prostate cancer treatment can cause adverse effects, such as incontinence or erectile dysfunction, which affect the patient’s quality of life. Therefore, it needs careful consideration, as sometimes the risks outweigh the benefits of being treated [58]. For tumors found in the early stages, which show a slow progression, watchful waiting is an option for patients whose life expectancy is less than five years. Active surveillance is recommended for low-risk cancers which have not been spread beyond the prostate and have a Gleason score of up to six. They will undergo periodic PSA-level testing, digital rectal examinations, and biopsies at specific intervals [59].

Local treatment options include surgical approaches, focal therapies, and radiotherapy. Surgical removal of the prostate can be done either laparoscopically or in open surgery. Open retropubic or perineal radical prostatectomy was commonly used until laparoscopic and robotic-assisted radical prostatectomies became routine surgeries for prostate cancer-diagnosed patients. Transurethral resection of the prostate (TURP) is also a viable option for symptom management but not for treating cancer, as it removes prostate tissue to relieve some urinary manifestations, such as nocturia and infections. It is therefore reserved for adenomas [60].

Interestingly, despite the type of procedure done, the risk and severity of adverse effects of a prostatectomy seem to remain the same, with urinary incontinence and sexual dysfunction affecting the quality of life [61]. Focal therapies include cryoablation and high-intensity focused ultrasound (HIFU), which can remove small, low-risk tumors from the prostate but are still a topic of several studies, to determine whether they are as effective as surgery or radiation therapy [62]. Radiation treatment can be external-beam radiation therapy or brachytherapy, in which the radioactive source is implanted into the prostate, affecting a smaller amount of healthy tissue. Proton therapy has not been proven to be a more viable option for prostate cancer treatment. Radiotherapy can cause urinary and bowel dysfunctions and sexual dysfunction [39, 40].

Systemic treatment can be administered individually or as part of a treatment plan, including surgery or radiation therapy. One of the essential systemic treatments is hormone therapy, which aims to lower sex hormone levels and thus block the AR pathway. Most prostate cancers are androgen-dependent, so low androgen levels should significantly slow tumor growth [63]. Castration can be surgical by performing bilateral orchiectomy and thus stopping androgen production in the testicles, or chemical, by administering a medication that blocks the AR pathway. Chemical castration is often reversible [64]. Luteinizing hormone-releasing hormone (LHRH) agonists and antagonists effectively reduce androgen production in the testicles. Androgen production in the adrenal gland or even some prostate cells can be blocked with androgen synthesis inhibitors, such as ketoconazole or abiraterone acetate, which is a CYP17A1 inhibitor and affects testosterone production from progesterone. However, ketoconazole is no longer widely used due to its many drug interactions [42]. AR inhibitors, such as enzalutamide, block androgens binding to the corresponding receptors and therefore prevent them from exerting their effects [43]. Hormone therapies have several significant side effects which affect sexual function, cause weight gain, gynecomastia, cardiovascular involvement, or even depression and memory impairment [42]. An important issue for patients treated with hormone therapy is bone health. In many cases, they can develop osteopenia or osteoporosis. Bone-modifying drugs, such as zoledronic acid, alendronate, or risedronate, can be administered to those at high risk of developing lower bone density. These drugs do not seem to prevent bone metastasis of prostate cancers, however, they have a role in preventing complications related to bone health in patients who already have metastatic tumors in the bones [65]. Around 10%–20% of prostate cancers develop mechanisms that help them progress despite low testosterone levels, including CRPCs [39]. Bipolar androgen therapy (BAT) is a new treatment for men whose prostate cancer has become resistant to standard hormone-blocking therapy [66] and involves administering high levels of testosterone. The mechanism of BAT is based on excessive testosterone ability to induce DNA damage, activate cell stress pathways, stimulate immune-activating proteins, and even deregulate the activity of growth-promoting genes. Recent clinical studies revealed that BAT could be safely administered to asymptomatic patients with metastatic CRP, inhibits disease progression, produces sustained PSA in 30%–40% of patients, and resensitizes and prolongs response to subsequent antiandrogen therapy [67].

Chemotherapy is used to treat advanced, metastatic prostate cancers. Unfortunately, the well-known side effects seriously affect the patient’s quality of life. Standard chemotherapy regimens start with docetaxel, a taxane that inhibits microtubular depolymerization. Taxanes are the only chemotherapeutic agents with demonstrated benefits on the survival of patients with metastatic CRPCs [68]. Although platinum-based chemotherapy is not widely used in prostate cancers, studies showed that patients with mutations that alter DNA damage repairs, such as defective mismatch repair in Lynch syndrome or defects caused by *BRCA2* mutations, respond well to platinum-based cytostatic drugs. However, multiple studies are needed to determine if platinum-based chemotherapy should be a standard approach in prostate cancer treatment for patients with specific genetic findings [69]. Most patients present with prostate adenocarcinoma, an androgen-driven luminal type tumor typically associated with elevated PSA and are sensitive to AR inhibition therapy. However, poorly differentiated neuroendocrine carcinomas (NEPC) similar to small-cell carcinoma histology are more aggressive and require platinum-based chemotherapy using small-cell-lung carcinoma (SCLC) regimens [70]. About 20% of patients treated with hormonal therapies develop small-cell carcinomas as a resistance mechanism (t-NEPC) and coexist with adenocarcinoma as a heterogenous mixt tumor. It is currently updated in the National Comprehensive Cancer Network (NCCN) guidelines to include consideration of metastatic biopsy and subsequent *RB1* and *TP53* somatic analysis [71].

Using iron as a complementary treatment measure for antiandrogenic therapy seems to increase the efficacy of prostate cancer cell death. Iron was proven toxic for cancer cells, causing oxidative stress and protein damage [72].

Current treatment options for bone metastases which occur in more than 60% of patients with advanced prostate cancer are palliative and not curative, therefore, the search for potential therapeutics is stringent, but still challenging [73]. Bisphosphonate and denosumab reduce bone fragility and delay metastases progression. Tyrosine kinase inhibitors like cabozantinib (XL184) and dasatinib have also been investigated in bone metastasized prostate cancer with promising outcomes in clinical trials. Atrasetan, an endothelin-A receptor (ETAR) antagonist, lowers the level of PSA and the incidence of bone pain in metastatic patients [74].

Novel treatment options in oncology target-specific disease progression mechanisms, offering both better results and limited impact on healthy cells. Genetic testing is essential in finding the appropriate targeted therapy based on each patient’s specific cancer and molecular characteristics [75]. Anti-PD1 antibody therapy appears to be effective in a limited number of prostate cancers in which defective mismatch repair and microsatellite instability have been demonstrated, but the results are not consistently promising. However, it is notable that *CDK12* mutated cell lines showed favorable responses to anti-PD1 therapy [76]. Patients with metastatic CRPCs, who do not respond to AR inhibitors and androgen synthesis inhibitors and have DNA repair defects, are candidates for targeted therapy with PARP inhibitors. Currently used PARP inhibitors are olaparib or rucaparib, which are effective in particular prostate cancer cases [77]. The role of PARPi is demonstrated especially in treating cancer cells with defective homologous recombination, which are susceptible to the alteration of the base excision repair pathway. The most important example of such cell lines is *BRCA*-mutated cancer cells. *TMPRSS2-ERG* fusion, *PALB2* mutations, and *PTEN* loss also make the cell lines good candidates for PARPi therapy. On the other hand, *ATM, CHEK2,* and *CDK12* mutations were associated with lower response to PARP inhibitor treatment [78].

Immunotherapy has not been of great importance in the treatment of prostate cancer and has not significantly improved clinical outcomes. Immunotherapy with sipuleucel-T, an active cell-based autologous immunotherapy, has been attempted for metastatic prostate cancer. Although it does not lead to tumor shrinkage or lower PSA levels, it prolongs patients’ life by up to four months in advanced diseases. Immunotherapy in prostate cancer can be used only if patients are asymptomatic. However, its benefits could not be clearly stated. Although showing principal activity, sipuleucel-T is no longer used in routine practice [79]. Vaccination is not yet available in clinical practice. PROSTVAC, a PSA recombinant vaccinia vector, showed promising activity in metastatic CRPC patients [80].

PSMA levels are much increased in cancerous cells compared to healthy prostate tissue. PSMA represents an important diagnostic tool and can also be the target of a humanized monoclonal antibody, J591, for metastatic prostate cancer patients. It remains to be studied how to correctly select the target groups which qualify for this novel treatment. PSMA-antibody-drug conjugates and PSMA-based chimeric antigen receptor-therapy (CAR)-T is the subject of several studies which target PSMA as a treatment option for CRPC [81]. PSMA BiTE is a bispecific CD3 and PSMA antibody construct, which re-directs and activates T-cells to PSMA-expressing cells already used in treating other malignancies and showing promising results in prostate cancer [82]. In clinical trials, three other bispecific T-cell engagers, glypican-1 and disintegrin and metalloproteinase 17 (ADAM 17) and six-transmembrane epithelial antigen of the prostate 1 (STEAP-1) are under evaluation [83–85].

*TP53* inactivation plays an important role in second-generation anti-androgens drug resistance and neuroendocrine differentiation, therefore, tumor suppressor reactivation by blocking the interaction of MDM2-p53 is a promising target in metastatic CRPC [86]. Idasanutlin is currently the subject of several ongoing clinical trials. It targets *p53* altered cell lines, which in case of prostate cancer, together with alterations in *RB1,* leads to castration-resistant, aggressive tumors with a poor prognosis. However, available studies referred to hematological malignancies, and clinical trials specific to prostate cancers are needed in the future [87].

As *AKT* is a crucial component of the PI3K pathway, PI3K inhibitors demonstrated only partial responses in some early-phase clinical trials [7]. AKT inhibitors that reach the clinical trial phase can be either ATP-competitive inhibitors, such as ipatasertib or capivasertib, or allosteric inhibitors, such as perifosine or MK-2206. Ipatasertib showed some prolonging of the progression-free interval in some phase 2 trials. The combination of docetaxel and capivasertib is the subject of ongoing clinical trials. Ipavasertib and abiraterone, administered in combination, resulted in significantly better progression-free survival, demonstrated radiologically [88]. However, several studies are needed before their potential inclusion in clinical practice as treatment options for prostate cancers [89].

**Figure 5.**
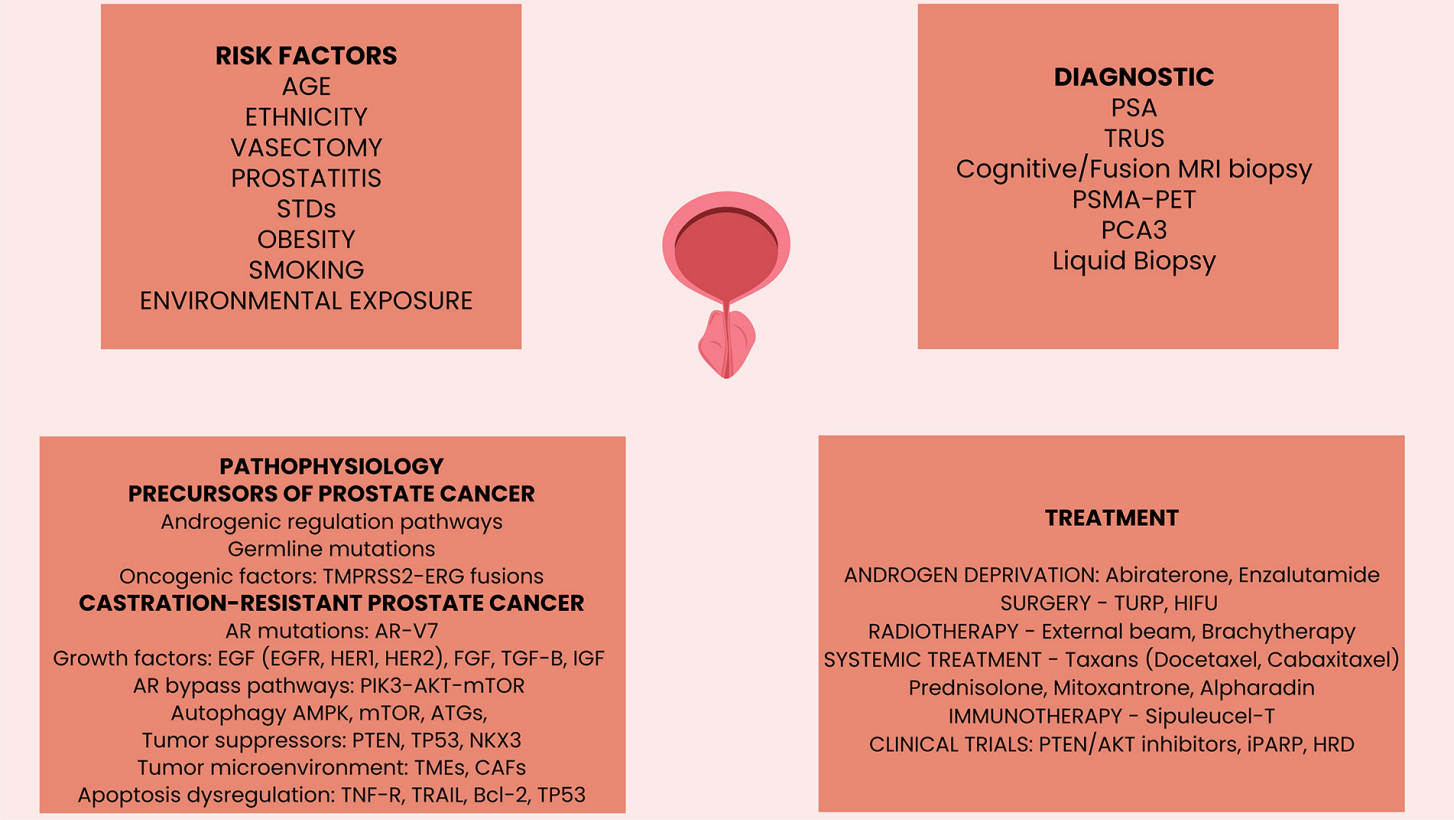
**Graphical abstract.** AR: Androgen receptor; AR-V7: Androgen receptor splice variant 7; AMPK: 5'AMP adenosine monophosphate-activated protein kinase; ATGs: Autophagy-related genes; Bcl-2: B-cell lymphoma; CAFs: Cancer-associated fibroblasts; EGF: Epidermal growth factor; EGFR: Epidermal growth factor receptor; HER: Human epidermal growth factor receptor; HIFU: High intensity focused ultrasound; IGF: Insulin growth factor; MRI: Magnetic resonance imaging; NKX3-1: NK3 Homeobox 1; iPARP: Poly (ADP-ribose) polymerase inhibitors; PCA3: Prostate cancer antigen 3; PIK3-AKT-mTOR: Phosphatidylinositol 3-kinase/mammalian target of rapamycin pathway; PTEN: Phosphatase and tensin homolog; PSA: Prostate specific antigen; TRUS: Transrectal ultrasound scan; PSMA-PET: Prostatic specific membrane antigen-positron emission tomography; TMPRSS2-ERG: Transmembrane protease serine 2 v-ets to erythroblastosis virus E26 fusion; TGF-β: Transforming growth factor beta; TMEs: Tumor microenvironment immune elements; TNF-R: Tumor necrosis factor receptor; TP53: Tumor suppressor protein 53; TRAIL: Tumor necrosis factor-ligand apoptosis-inducing ligand; TURP: Transurethral resection of the prostate.

## Genetic counseling

Given the importance of genetic testing in the clinical management of several pathologies, genetic counseling has a rising role in patient care. For example, in case of prostate cancer, it aims to assess hereditary cancer risk, recommend genetic testing for the patients that could benefit from it, and explain all the possible outcomes of undergoing genetic testing so that the patient can make a fully informed decision. Furthermore, once the genetic test is performed, medical geneticists should interpret the results with the patient and explain what they imply for the patient and his family members, who might also need further investigations to assess their hereditary cancer risk [90]. The results of any genetic testing must be interpreted, considering the personal and family history previously discussed with the patient. Genetic testing might guide the treatment plan for prostate cancer, as some mutations make the patient a good candidate for targeted therapy, thus offering better outcomes and minimizing the side effects of the treatment [91]. Some of the obtained results might require further investigations or research, and the patient must be informed about these possibilities before the testing. Such results can be variants of uncertain significance (VUS) or even negative results in patients with a significant family history of cancers [43, 92]. The psychological impact of being diagnosed with cancer is significant, with psychological support being a crucial part of patient care. Hereditary cancers imply an even more significant impact, as family members might also be affected [93]. Germline mutations in *BRCA1* and *BRCA2* genes were reported in about 15% of metastatic prostate cancers [94, 95]. Detecting germline mutations in prostate cancer is important from the perspective of oncological risk assessment, personalized therapy, and family genetic counseling. According to the NCCN guidelines (Version 1.2021) [96], germline molecular testing is recommended for men with any one of the following: family history of a relative with a germline mutation, metastatic prostate cancer, regional or node-positive disease, high-risk prostate cancer (defined by grade group, T staging, and PSA levels at diagnosis), intraductal or cribriform histology, and Ashkenazi Jewish ancestry. All men carrying *BRCA1* or *BRCA2* mutations should start cancer screening at the age of 40 with yearly PSA and consider the same in men with *BRCA1*, *ATM*, *HOXB13*, and DNA MMR mutations. Also, men with metastatic CRPC and mutations in DNA repair genes, such as *BRCA1*, *BRCA2*, and *ATM* might indicate PARPi therapy. Prophylactic prostatectomy is not indicated for mutation carriers [97].

## Conclusion

Prostate cancer significantly contributes to increased cancer-related mortality rates in men globally. Although most cases have an early diagnosis, the disease invariably evolves toward advanced disease. Periodic PSA-based screening remains the most commonly used screening method for early detection of prostate cancer, closely followed by diagnostic TRUS, MRI, and PSMA-positron emission tomography (PSMA-PET). Radical prostatectomy or ablative radiotherapy are curative approaches in localized cases. In relapsed cases, radiotherapy or androgen deprivation therapy combined with chemotherapy or novel androgen signaling-targeted agents are used to control the systemic disease evolution. CRPC is targeted with AR agents, chemotherapy, radionuclides, and PARPi. Despite therapies that have improved survival rates, metastatic prostate cancer remains incurable. Even if prostate cancer does not have molecular characteristics that would make it an ideal candidate, immunotherapy remains to be an engaging treatment option for prostate cancer (Figure 5).
